# Nanoquercetin based nanoformulations for triple negative breast cancer therapy and its role in overcoming drug resistance

**DOI:** 10.1007/s12672-024-01239-y

**Published:** 2024-09-17

**Authors:** Adyasa Samantaray, Debasish Pradhan, Nalini Ranjan Nayak, Saurabh Chawla, Bandana Behera, Lalatendu Mohanty, Saroj Kanta Bisoyi, Sabnam Gandhi

**Affiliations:** 1 University Department of Pharmaceutical Sciences, Utkal University, Vani Vihar, Bhubaneswar, Odisha India; 2 School of Biological Sciences, National Institute of Science Education and Research (NISER) Bhubaneswar, Khurda, Odisha India; 3 Faculty of Pharmacy, C.V.Raman Global University, Bhubaneswar, India; 4 Department of Pharmaceutical Sciences, HNB Garhwal University, Uttarakhand, India

**Keywords:** Nano-quercetin, Anticancer, Chemotherapy resistance, Nano medicine

## Abstract

Triple Negative Breast Cancer (TNBC) is a highly aggressive and treatment-resistant subtype of breast cancer, lacking the expression of estrogen, progesterone, and HER2 receptors. Conventional chemotherapy remains the primary treatment option, but its efficacy is often compromised by the development of drug resistance. Nanoquercetin has garnered the attention of researchers due to its potential in combating cancer. This antioxidant exhibits significant efficacy against various types of cancer, including blood, breast, pancreatic, prostate, colon, and oral cancers. Functioning as a potential anti-cancer agent, nanoquercetin impedes the development and proliferation of cancer cells, induces apoptosis and autophagy, and prevents cancer cell invasion and metastasis. Numerous processes, such as the inhibition of pathways linked to angiogenesis, inflammation, and cell survival, are responsible for these anticancer actions. Moreover, it shields DNA from degradation caused by radiation and other carcinogens. The cost-effectiveness of current cancer treatments remains a significant challenge in healthcare, imposing a substantial economic burden on societies worldwide. Preclinical studies and early-phase clinical trials indicate that nanoquercetin-based therapies could offer a significant advancement in the management of TNBC, providing a foundation for future research and clinical application in overcoming drug resistance and improving patient outcomes. This article examines the latest data on nanoquercetin’s potent anti-cancer properties and interprets the accumulated research findings within the framework of preventive, predictive, and personalized (3P) medicine.

**Graphical Abstract:**

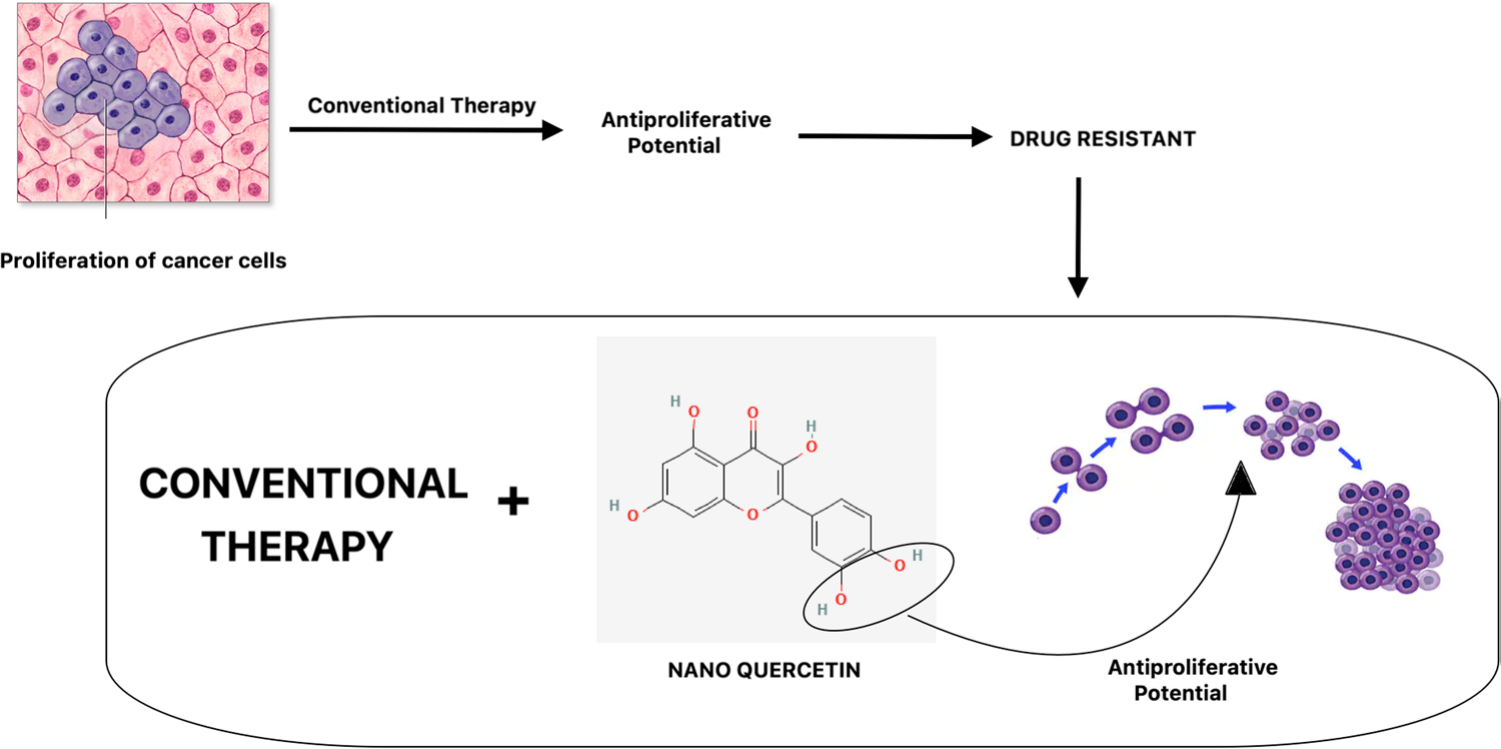

## Introduction

Pharmaceutical nanotechnology-based drug delivery systems are conveyance vehicles for active pharmaceutical ingredients (APIs) that are targeted and delivered. These nanosystems, which are already available on the market, primarily consist of anticancer medications. Their nanoscale size offers various advantages over conventional liquid and solid dosage forms such as suspensions, tablets, capsules, and emulsions [1]. These advantages stem from their physicochemical properties, particularly their size and composition, including lipids, surfactants, and amphiphilic block copolymers. Numerous of these systems allow for controlled release of hydrophobic and hydrophilic APIs and are biocompatible [2]. They can also improve the encapsulated APIs’ Absorption, Distribution, Metabolism, Excretion, and Toxicology [ADME(T)] profile [3]. There are a number of ways to give nanoformulations, including transdermal, nasal, intramuscular, and intravenous (IV) methods. Notably, nanocarriers offer advantages in targeting specific tissues and overcoming physical barriers. However, designing and developing nanoformulations present challenges. Complete characterization requires multiple techniques, increasing development costs compared to conventional formulations. The pharmaceutical sector has challenges due to the high cost of nanomaterials, along with regulatory uncertainties and limited understanding of nanomedicine performance in humans.

According to the WHO, cancer is the leading cause of death, and breast cancer is projected to continuously rise in the number of deaths. Cancer can develop due to an incorrect diet, genetic predispositions, and environmental issues. Chemotherapy is used and administered in repeated cycles for treatment [4]. Chemotherapy drugs are toxic, and there is no clear way for efficient, targeted delivery of these drugs. Chemotherapy is usually given in repeated cycles of treatment. Thus, conventional approaches have demonstrated limited success in the treatment and cure of cancer. Treating cancer with conventional therapeutics, such as chemotherapy and radiation, faces significant challenges due to drug resistance. Cancer cells can exhibit intrinsic resistance through mechanisms like drug efflux pumps, enhanced DNA repair, and mutations in drug targets. They can also acquire resistance during treatment via genetic mutations, epigenetic changes, and cellular adaptations. These resistances, combined with tumor heterogeneity, toxicity, metastasis, and the tumor microenvironment, complicate treatment. Strategies to overcome these challenges include combination therapy, targeted therapy, immunotherapy, personalized medicine, nanotechnology, and epigenetic therapy. These approaches aim to enhance efficacy, reduce side effects, and address resistance mechanisms. For these reasons, more effective alternative cancer therapeutic compounds with minimal side effects are desperately being sought. Natural products with nanoformulations have exhibited pharmacological and biological properties that hold promise for disease treatment. They also serve as inspiration for potential new medications.

**Fig. 1:**
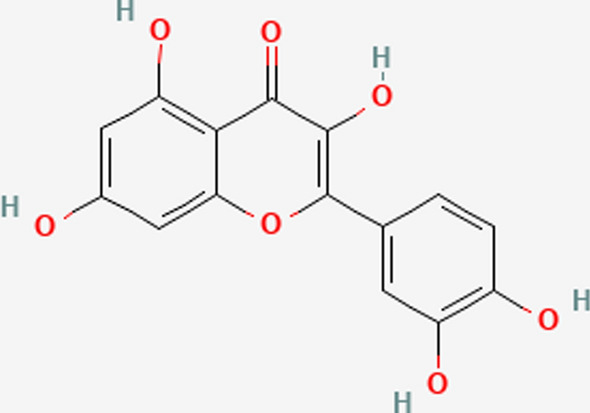
Structural view of quercetin (Chemical structure)

Extensive studies have been conducted in the previous few decades on natural products, exploring their applications in medicinal chemistry, molecular science, and pharmaceuticals. Quercetin, derived from the Latin word “quercetum” meaning plantation of oak trees, is a prominent flavonoid abundantly present in a variety of plant sources such as vegetables, fruits, and beverages. It constitutes a significant portion of the human diet, with approximately 60–75% of total flavonoid intake attributed to it. Structurally, quercetin belongs to the class of 3-hydroxyflavones (flavonols) and exhibits a yellow color in its powdered form. Its chemical composition includes hydroxyl groups at specific positions, influencing its pharmacological activities and potential derivatives as shown in Fig. 1. Quercetin, a well-known flavonoid, possesses notable pharmacological properties including anti-inflammatory, antioxidant, and anticancer effects [5]. Unfortunately, its limited absorption and solubility limit its therapeutic relevance. To address this limitation, numerous studies have explored various nanosystems aimed at improving the bioavailability and efficacy of quercetin. A viable method for creating quercetin dosage forms with improved bioavailability for oral and other modes of administration is provided by these nanosystems [6, 82].

In order to encapsulate quercetin, target particular tissues, and achieve regulated release, advanced delivery systems are utilised. These systems include nanoparticles, liposomes, nanoemulsions, and nanocrystals, among others. Nanoparticles, such as solid lipid nanoparticles and polymeric nanoparticles, provide a means to encapsulate quercetin and protect it from degradation, thereby enhancing its stability and bioavailability [7, 83, 84]. Liposomes, which are lipid-based vesicles, offer a versatile platform for delivering quercetin and can be tailored to improve its solubility and target specific tissues. Nanoemulsions are another effective delivery system that can encapsulate quercetin within nanoscale droplets, enhancing its solubility and facilitating absorption. Nanocrystals represent a strategy to increase the bioavailability and rate of dissolution of quercetin by reducing its particle size to the nanometer range [8, 85].

Nanoquercetin, a nanotechnology-enhanced formulation of quercetin, addresses the limitations of pure quercetin by improving its solubility, bioavailability, and stability. This advanced formulation protects quercetin from degradation, allows for controlled and sustained release, and enables targeted delivery to specific tissues, thereby enhancing its therapeutic efficacy and reducing side effects. Consequently, nanoquercetin requires lower doses to achieve desired effects, making it a more potent and practical option for clinical use, particularly in its antioxidant and anti-inflammatory roles.

### Nano-quercetin

Quercetin, a bioactive flavonoid, possesses strong antioxidant properties because of its capacity to bind transition metal ions and scavenge free radicals.Its antioxidant activity stems from phenolic hydroxyl groups, including those in the B ring o-dihydroxyl groups, 4-oxo groups, and 3- and 5-hydroxyl groups [9]. These functional groups donate electrons to stabilize radicals, making quercetin a potent antioxidant. In addition to its antioxidant effects, quercetin exhibits anti-obesity, anti-diabetic, anti-inflammatory, anti-hypertensive activities, etc. [10]. However, due to its low bioavailability, its health benefits are limited, typically less than 2% of the administered dose.

To overcome this limitation, novel strategies based on nanotechnology have been created to enhance the bioavailability of quercetin. These are lipid based nanosystems, nanovesicles, thermoresponsive mesoporous silica nanoparticles, nanosuspension, polymeric nanoparticles, etc. When compared to pure quercetin powders, quercetin-loaded solid lipid nanoparticles have demonstrated noticeably better bioavailability. In animal studies, these nanoparticles have shown antioxidant qualities akin to those of free quercetin, such as streptozotocin-induced diabetic rats. Furthermore, quercetin self-emulsifying nanoformulations have demonstrated greater antioxidant capability than quercetin in its free form, in combating cardiotoxicity and nephrotoxicity induced by doxorubicin and cyclosporine A, respectively [11]. Quercetin-loaded nanorods and silica nanoparticles have also shown promising results in enhancing antioxidant defense mechanisms and ameliorating inflammatory conditions in various cell lines and animal models. It is commonly acknowledged that CHSNPs can enhance the therapeutic efficacy of hydrophobic medications while reducing their adverse effects. Their polycationicity, bioactivity, biodegradability, and biocompatibility are the causes. Moreover, the CHSNPs demonstrate drug targeted delivery, solubilize a range of hydrophobic medications, improve blood circulation and bioavailability, improve encapsulation effectiveness, and maintain drug release. To create CHSNPs, a mixture containing 50 millilitres of 1% acetic acid, 500 milligrammes of 85% deacetylated chitosan, and 1 milligramme of TPP per millilitre was agitated at 1400 rpm for thirty minutes.

These findings suggest the potential of nanotechnology-based formulations to enhance the therapeutic efficacy of quercetin and mitigate its limitations in clinical applications.

### Therapeutic advantages of nano-delivery systems

**Fig. 2:**
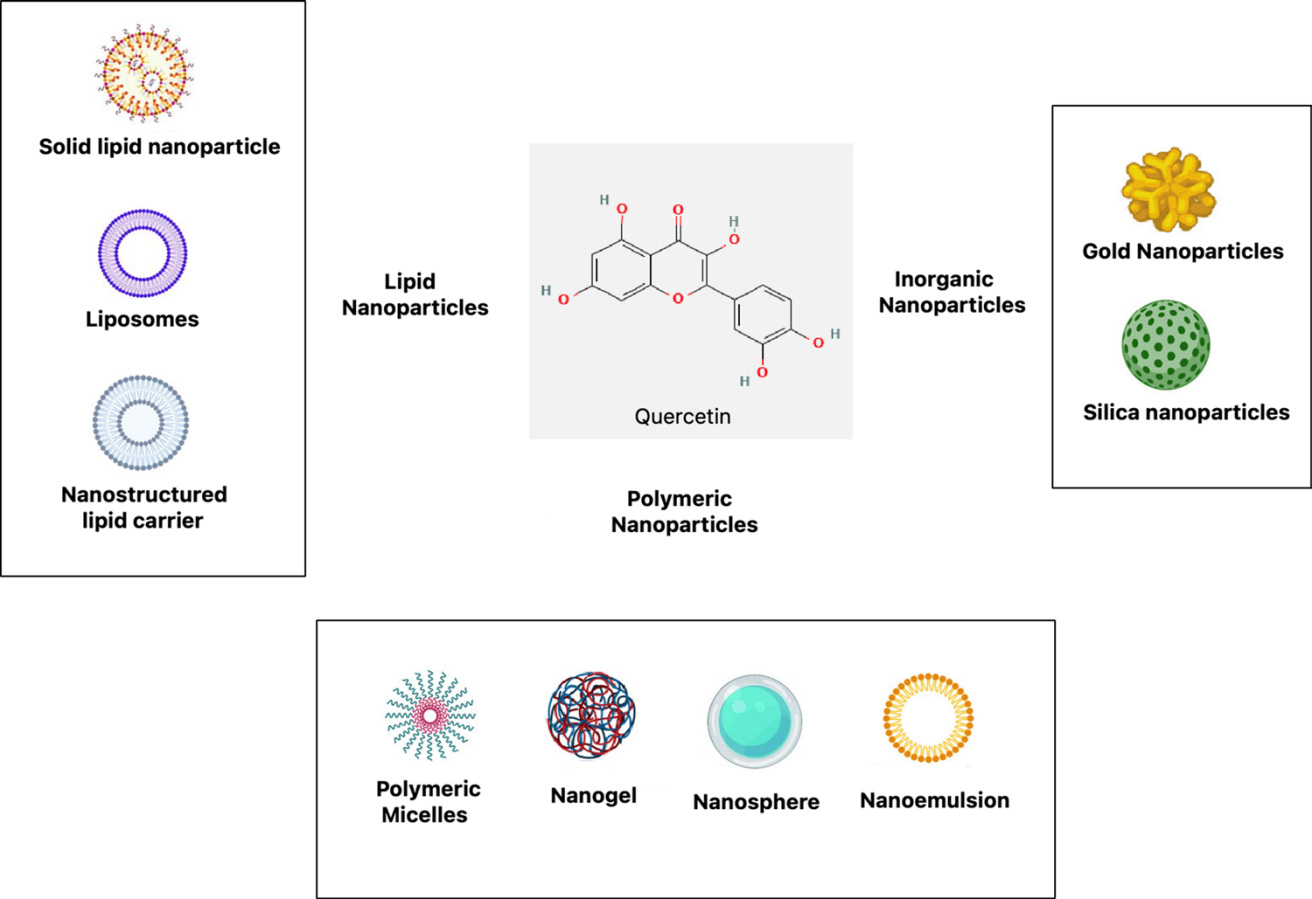
Nanoformulations to carry quercetin, a variety of nanocarriers

Therapeutic targeting using nanostructures might be passive or active. The delivering nanovector erodes or diffuses, releasing the therapeutic substance in passive nanodelivery. On the other hand, active delivery uses RNAs, proteins, lipids, carbohydrates, and tiny metabolites as biomarkers to allow for the regulated release of biological substances at specific locations in the body [12]. Selective targeting to bodily areas or tissues induced by heat, ultrasound, electric or magnetic fields, light, pH changes, or exposure to specific enzymes is made possible by the incorporation of stimuli-responsive components. Metallic nanoparticles like silver, gold, and iron oxide can also be modified to act as drug carriers. However, organic nanocarriers are preferred due to their tunable physicochemical properties via modification of size, morphology, composition, shape, and surface characteristics. The efficiency of nanodelivery depends on the molecular weight of the therapeutic agents, with higher molecular weight compounds showing lower bioavailability [13]. Different therapeutic nanodelivery systems have different features as Fig. 2 and Table 1 depicts, loaded agents, and therapeutic applications that contribute to their health benefits. Compared to conventional formulations, natural compound-loaded nanoparticles have better tissue targeting, permeability, stability, half-life, solubility, and bioavailability. They also have fewer negative effects. Systems based on nanotechnology are being utilised more and more to prevent and treat diseases like tumours, obesity, diabetes, cardiovascular disease, and neurodegenerative disorders that are linked to ageing.

**Table 1:** Various nanoformulation of quercetin and its applications [14–20]

Formulations	Applications
Oleogels	Oleogels, with particle sizes ranging from 345.3 to 401.5 nm, improve skin permeation and are evaluated using ex-vivo and in-vitro methods
Nanovesicles	Nanovesicles, fabricated using the thin-film hydration technique, have particle sizes between 125 and 184 nm and are used for anti-acne applications. These are evaluated through ex-vivo and clinical studies
Chitosan tripolyphosphate nanoparticles	Chitosan tripolyphosphate nanoparticles, created via the ionic gelation method, have a particle size of 361.16 nm and are employed for cutaneous wound healing, with evaluations done in vivo
Transfersomes	Transfersomes, developed using the conventional thin-film hydration method, have a particle size of 75.95 nm and are used for treating secondary osteoporosis, with evaluations done ex-vivo and in-vitro
Hydrogels	Hydrogels made using an in-house developed method serve anti-inflammatory, anti-aging, and skin permeation improvement purposes, with evaluations conducted ex-vivo and in-vitro
Gelatin films	Gelatin films, produced through solvent evaporation, have a particle size of 160 nm and exhibit anti-oxidant properties, evaluated in-vitro
Hydrogel	Hydrogels fabricated through a low-energy spontaneous emulsification method are used for diabetic wound healing and evaluated in vivo
Nanoemulsion based gel	Nanoemulsion-based gels, created by spontaneous emulsification, with particle sizes ranging from 125.4 to 45.4 nm, are applied for rheumatoid arthritis and evaluated in-vivo and in-vitro
Nanogel	Nanogels, made by high-pressure homogenization with particle sizes of 249.65 and 352.48 nm, are used for anti-cancer applications and evaluated in vivo
Gold nanoparticles	Gold nanoparticles, incorporating citrate groups as capping elements and having particle sizes ≤ 100 nm, have anti-oxidant, antimicrobial, and cytotoxic properties, evaluated in-vitro
NLC	NLCs (Nanostructured Lipid Carriers), developed through emulsion and sonication methods with particle sizes of 130 nm, have anti-oxidant, anti-allergic, photoprotective, and skin permeation improvement properties, evaluated in vitro
Micelles	Micelles, created via cationic ring-opening polymerization with a particle size of 19 nm, exhibit anti-oxidant properties and are evaluated in-vitro
Modified collagen hybrid scaffold	Modified collagen hybrid scaffolds, produced by ultrasonication with particle sizes ranging from 300 to 500 nm, are used for diabetic wound healing and evaluated in-vivo and in-vitro
Lipid nanocapsules, Liposomes, and smart Crystals^®^	Lipid nanocapsules, liposomes, and smart crystals, fabricated using ethanol injection and phase inversion techniques with particle sizes of 26 to 295 nm, improve skin delivery and are evaluated in-vitro
Multiphase hydrogel	Multiphase hydrogels are used for wound healing and evaluated both in-vitro and in-vivo
Microemulsions	Microemulsions, created by the water titration method with a particle size of 25 nm, provide anti-oxidant and UV protection and are evaluated in-vitro and ex-vivo
Hydrogel	Hydrogels are used to improve skin permeation and are evaluated ex-vivo
Calcium phosphate- nanocomposite	Calcium phosphate-nanocomposites, created by precipitation methods with particle sizes of 460 and 497 nm, exhibit anti-oxidant properties and are evaluated in-vitro
Lipid nanocapsules	Lipid nanocapsules, produced using the phase inversion technique with particle sizes of 50 and 20 nm, provide anti-oxidant and cellular protection and are evaluated in-vitro
Microemulsions	Microemulsions, with particle sizes ranging from 9.74 to 12.66 nm, improve skin permeation and are evaluated through in-vitro skin permeation studies
Smart crystals®	Smart crystals, developed by wet bead milling and high-pressure homogenization with particle sizes ranging from 303 to 574 nm, exhibit anti-oxidant properties and are evaluated in-vitro
Silica nanoparticles	Silica nanoparticles, created by copolymerization with particle sizes of 3.5 nm and 5.0 nm, have anti-oxidant properties and are evaluated in-vitro and ex-vivo
Self-double emulsifying drug delivery systems	Self-double emulsifying drug delivery systems, fabricated through a two-step emulsification process with particle sizes ranging from 3.66 to 25.32 nm, improve skin permeation and are evaluated in-vitro
Mesoporous silica nanocarriers	Mesoporous silica nanocarriers, created by the impregnation method with particle sizes of 250 nm, are used for anti-cancer applications and are evaluated in-vitro and ex-vivo
SLN	SLNs (Solid Lipid Nanoparticles), produced using homogenization and ultrasonication methods with particle sizes ranging from 274.0 to 986.6 nm, improve skin permeation and are evaluated in-vitro
Nanovesicles	Nanovesicles, fabricated using an ultrasonic disintegrator with particle sizes around 80 to 110 nm, are used for wound healing and evaluated in-vitro
NLC	NLCs, developed through probe ultrasonication with particle sizes of 282 nm, exhibit anti-oxidant and skin permeation properties and are evaluated in-vitro
SLN	SLNs, created using probe ultrasonication methods with particle sizes ranging from 26 to 155 nm and 97 to 349 nm, have anti-oxidant and skin permeation properties and are evaluated in-vitro
Biocompatible nanoparticles	Biocompatible nanoparticles, produced through desolvation-solvent evaporation with particle sizes ≥ 200 nm, exhibit anti-oxidant and anti-cancer properties and are evaluated in-vitro and ex-vivo
NLC	NLCs, fabricated using emulsion evaporation-solidification with particle sizes of 215.2 nm, exhibit anti-oxidation, anti-inflammation, and skin permeation improvement properties, evaluated in-vivo and in-vitro
Lecithin-chitosan nanoparticles	Lecithin-chitosan nanoparticles, with particle sizes of 95.3 nm, improve skin penetration and are evaluated in-vitro and in-vivo

### Anti‑cancer therapy: from conventional to advanced approaches

The evolution of cancer treatment over the past century has seen remarkable advancements, from the development of radiotherapy and chemotherapy to the introduction of targeted therapies and immunotherapeutic agents [21]. These innovations have revolutionized oncology, offering more personalized and effective treatment options for cancer patients.

In recent decades, research has focused on developing anti-tumor strategies that target specific molecular alterations in tumors, such as monoclonal antibodies and immune checkpoint inhibitors. These targeted therapies have significantly improved treatment efficacy and reduced toxicity, leading to better outcomes for many patients.

However, a major challenge in cancer treatment is the emergence of resistance to therapy, which can lead to cancer relapse and recurrence [22]. Despite initial positive responses to treatment, many patients experience relapse due to acquired resistance mechanisms.

To address this challenge, precision medicine has become a viable strategy, allowing for individualized cancer treatment based on predictive and preventive strategies [23]. Precision medicine attempts to decrease the likelihood of resistance and enhance results by customising treatment regimens to the particulars of each patient's cancer.

Furthermore, natural substances have shown promise as adjunctive therapies in cancer treatment by sensitizing cancer cells to therapeutic agents [24]. Some compounds, such as quercetin, may overcome resistance mechanisms and increase the effectiveness of traditional cancer treatments [25].

Overall, the integration of precision medicine and natural substances into cancer treatment strategies holds great promise for improving outcomes and addressing the challenge of treatment resistance in cancer patients. Continued research in these areas is essential to further advance cancer care and optimize patient outcomes.

### Nanoquercetin as a helper in anti-cancer therapy

These advanced delivery systems enable precise control over the release of quercetin, allowing for sustained and targeted delivery to the desired site of action [26]. Additionally, they can improve the stability and solubility of quercetin, thereby enhancing its therapeutic efficacy [27]. Overall, the development of nanosystems for quercetin delivery holds great promise for overcoming its limitations and expanding its clinical applications.

The review highlights quercetin's potential to enhance the effectiveness of conventional cancer treatments by overcoming therapy resistance in cancer cells. Specifically, quercetin's ability to sensitize malignant cells to various cancer therapies is of significant interest, as demonstrated in preclinical studies. These findings suggest that quercetin could have substantial clinical utility in personalized cancer treatment approaches as depicted in Table 2.

**Table 2:** Potential therapeutic effects of quercetin on various cancer. [25, 28–36]

Cancer Type	Dose	Model (Invitro/invivo/human)	Cell line/animal/human	Target	Result	Mechanism
Oral Cancer	10–100 μM,	Invitro	SCC25	Blc2, Bax, and caspase 3	cell cycle arrest in the G1 phase and triggered apoptosis in the mitochondria	decreased alterations in Hsp70 expression in EMT Increased apoptosis in cells resistant to drugs
Oral Cancer	100 mM	Invitro	SCC26	p38 MAPK–Hsp27, ABCG2, and MDR1	reduced development of tumours and medication resistance	decreased alterations in Hsp70 expression in EMT Increased apoptosis in cells resistant to drugs
Gastric Cancer	10–320 μM,	Invitro	AGS	Mcl-1, Bcl-2, Bcl-x, Bax, and MAPK	triggered cell death	decreased growth, decreased proliferation, suppression of the TRPM7 channel, and increased apoptosis
Gastric Cancer	380 nm	Invitro	AGS	CDC20-siRNA	Gastric Cancer cell development was inhibited	decreased growth, decreased proliferation, suppression of the TRPM7 channel, and increased apoptosis
Gastric Cancer	10, 15, and 20 μM	Invitro	MGC803	ROS-MAPK, P38, JNK, and ERK	triggered apoptosis and stopped the cell cycle in the G2/M phase	Increased programmed cell death Increased cell cycle arrest due to necrosis in G2/M phase
Gastric Cancer	20–100 μM	Invitro	MGC803	Bax, Bcl-2, cyt c, Oct4, Sox2, and CD44	apoptosis triggered by mitochondria	decreased apoptosis by a route that depends on mitochondria
Gastric Cancer	10 μM	Invitro	MGC803	V-FITC/PI, cyt c, ERK, and AKT	caused apoptosis and suppressed proliferation	Increased ROS; enhanced apoptosis; cell cycle arrest in the G2/M phase
Gastric Cancer	10, 15, and 20 μM	Invitro	SGC-7901	ROS-MAPK, P38, JNK, and ERK	triggered apoptosis and stopped the cell cycle in the G2/M phase	Decreased viability of the cell Increased ROS; enhanced apoptosis; cell cycle arrest in the G2/M phase
Colon cancer	160 μM	Invitro	SW480	Wnt	Cell cycle arrest in the G1/S phase	Enhanced β-catenin/Wnt decreased survivin, decreased cyclin D1,
Colon cancer	50 μM	Invitro	SW481	Wnt, β-catenin	Downregulated β-catenin, Tcf signaling	Enhanced β-catenin/Wnt decreased survivin, decreased cyclin D1,
Colon cancer	30 and 80 μM	Invitro	HT29	β-catenin	Antiproliferative effect	The amount of β-catenin in HT29 cells did not change
Esophageal cancer	0–10 μM	In vitro	SHEE and KYSE450	AKT/mTOR/p70S6K, and MAPK	anti-inflammatory, anti-proliferative, and tumour growth inhibition	slower growth Decreased rate of proliferation decreased inflammatory response decreased development of pre-neoplastic lesions by NMBA
Esophageal cancer	0–10 μM	In vivo	KYSE510	ERK, Ki67, c-Jun, and p-p70S6K	anti-tumor growth, anti-inflammatory, and anti-proliferation	slower growth Decreased rate of proliferation decreased inflammatory response decreased development of pre-neoplastic lesions by NMBA
Hepatocellular carcinoma	50 μM	In vitro	HepG2 Hep3B	Akt, pAkt, Bcl-2, caspase-3, and -9	triggered cell death	Decreased proliferation and growth increased cell cycle arrest during apoptosis in the G1 phase
Hepatocellular carcinoma	0.67 μM	In vitro	HepG2 Hep3B	–	minimally harmful effects on cancer cells and minimal antioxidant benefits	Decreased proliferation and growth increased cell cycle arrest during apoptosis in the G1 phase
Hepatocellular carcinoma	0–200 μM	In vitro and in vivo	LM3	JAK2 and STAT3	anti-migration, anti-invasion, antiproliferation, cell cycle arrest, and induced apoptosis	Decreased proliferation of tumour cells Decreased invasion and migration Increased autophagy in the S and G2/M stages of the cell cycle arrest
Leukaemia	0–200 μM	In vitro	HL60	pAkt, Bcl-2, BAX	Caused apoptotic processes through activating caspase families, raising BAX, and decreasing pAkt and Bcl-2 levels	Arrested cell cycle in G(0)/G(1) phase Increased apoptosis
Lungs Cancer	10–200 μM	In vitro	A549 H460	HSP70	Decreased HSP70 expression and decreased cell survival in both cell lines in a dose-dependent manner	Slower growth increased cell cycle arrest during apoptosis in the sub-G1 phase
Breast Cancer	0–250 mM	In vitro	− MCF 7	CDK6, ROS	caused apoptosis by reducing the expression of CDK6 and the generation of reactive oxygen species	Increased apoptosis decreased vitality of cancer cells
Prostate cancer	1, 20, 40, 800, 100, 200 mM	In vitro	LNCaP, PC-3, DU145, RWPE-1, HEK-293	–	Caused a notable reduction in cell viability that was dose-dependent. By blocking the cell cycle during the G0/G1 phase, it prevented the growth of prostate cancer cells. released caspase-3, poly ADP-ribose polymerase, and cytochrome c, which caused apoptosis	Decreased viability of the cell Increased cell cycle arrest during apoptosis in the G1 phase â† “cell migration
Cervical Cancer	25, 50, 100 μM	In vitro	HeLa	–	Anti-proliferation	Accelerated growth A rise in proliferation decreased establishment of colonies increased apoptosis More DNA damage to cells and cell cycle arrest in the G2/M phase Diminished migration of cells

By elucidating quercetin's mechanisms of action and its ability to modulate cancer cell responsiveness to therapy, researchers aim to identify strategies for optimizing cancer treatment outcomes. Quercetin’s role in sensitizing cancer cells to conventional treatments holds promise for improving therapeutic efficacy and overcoming resistance mechanisms.

In the context of personalized cancer therapy, quercetin's therapeutic potential lies in its ability to complement existing treatment modalities and address individual patient profiles. By incorporating quercetin into personalized treatment regimens, clinicians may enhance the effectiveness of cancer therapies and improve patient outcomes.

Overall, the review underscores the importance of quercetin as a potential adjunctive therapy in cancer treatment, particularly in overcoming therapy resistance and improving treatment response in personalized medicine approaches. Clinical trials and additional research are necessary to fully explore the clinical utility of quercetin in cancer therapy.

### Structural activity relationship of nanoquercetin with anticancer mechanism

Quercetin flavonoid, possesses a chemical structure characterized by a phenyl-substituted chromone framework comprising fifteen carbon atoms. This structure consists of the heterocyclic ring C and the benzo ring A produce the chromium nucleus, with a phenyl substitution in the aromatic ring B [37].

Studies indicate that the various substitutes present in rings A and B play crucial roles in determining the pharmacological activities of quercetin. However, there remains a lack of comprehensive data explaining the relationship between the chemical structure of quercetin and its cytotoxic and antitumor effects.

**Fig. 3:**
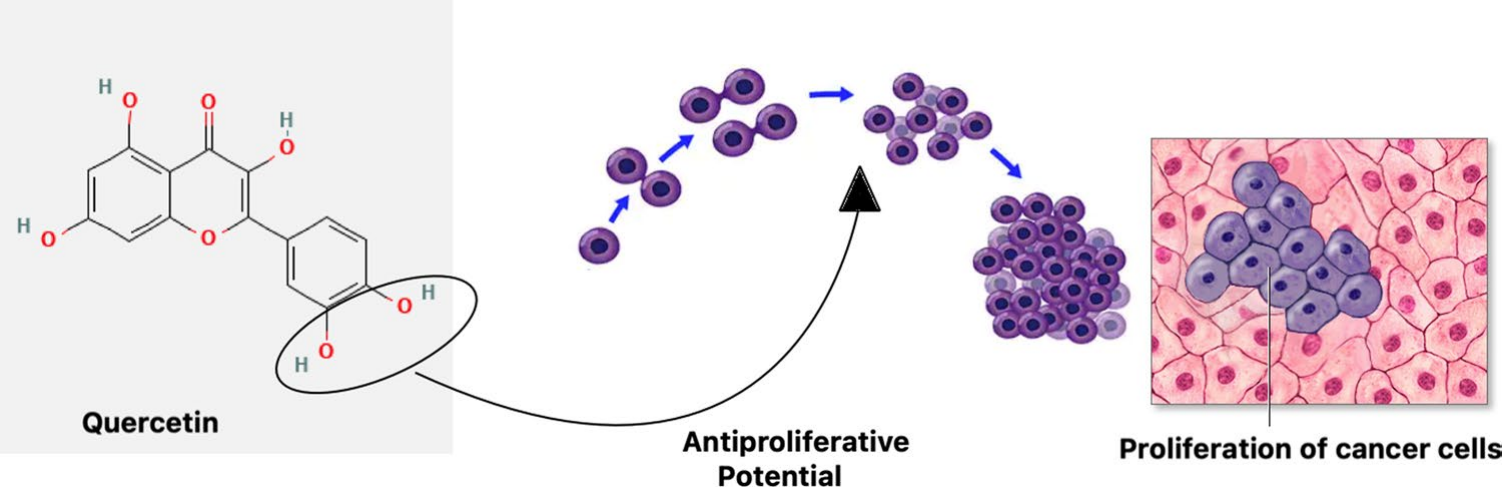
The potential structural activity relationship of nanoquercetin showing anti proliferative potential. Symbol: — > inhibition

The hydroxylation pattern of the B ring in quercetin's chemical structure is one possible correlation that has been proposed as the cause of the compound's antiproliferative properties and its ability to inhibit protein kinase B (AKT) as shown in Fig. 3. To fully understand quercetin's structure–activity interactions and their possible therapeutic applications for cancer, more investigation is required.

### Development and challenges of chemotherapeutic modalities

Chemotherapeutic modality development in the mid-twentieth century revolutionized cancer treatment, introducing various agents targeting cancer cell biology, such as DNA alkylating agents, antimetabolites, antimitotics, cytotoxic antibiotics, polyamine inhibitors, and iron-modulating drugs. Combination chemotherapy regimens were also developed to enhance efficacy.

#### Chemotherapy resistance

Despite their effectiveness, chemotherapeutic agents often face limitations due to primary or acquired resistance. Resistance mechanisms include increased drug efflux, variations in drug metabolism and signaling pathways, enhanced DNA repair, apoptosis evasion, mutations, reactivation of drug targets, interactions with the tumor microenvironment, and epithelial-mesenchymal transition (EMT). Cancer stem cells (CSCs) also play a role in resistance [38].

#### Multidrug resistance (MDR)

MDR is a significant challenge where cancer cells resist multiple unrelated agents. Key mechanisms involve increased drug efflux by proteins like ALDH and ABC transporters. Other factors include DNA repair mechanisms, genetic and epigenetic changes, xenobiotic metabolism, ER stress, and signaling pathways like PI3K/Akt and NF-kB [39].

#### Combating resistance

Recent research focuses on combining natural substances with chemotherapeutic actions, such as flavonoids, which have antioxidant, anti-inflammatory, and anticancer properties, making them ideal for combination therapy. Understanding chemotherapeutic resistance mechanisms and developing innovative strategies to combat it are crucial for improving cancer treatment and patient survival [40].

#### Targeted cancer therapy

Targeted cancer therapy, unlike conventional chemotherapy, focuses on specific molecular alterations in cancer cells, modifying processes like apoptosis induction, growth suppression, and metastasis restriction. This approach includes biological drugs like monoclonal antibodies (e.g., bevacizumab, cetuximab, pertuzumab, trastuzumab) and selective inhibitory compounds like tyrosine kinase inhibitors (TKIs) (e.g., imatinib, dasatinib, nilotinib, gefitinib, erlotinib, lapatinib, sunitinib, sorafenib) [30].

#### Challenges and advances

Resistance to single-agent targeted therapy often arises due to mutations or pro-survival signaling activation, unlike cytotoxic drug resistance caused by dysregulated pharmacokinetics. Combination therapies with next-generation medicines, such as flavonoids, may target resistance-associated mutations and pathways, offering a personalized approach to reducing targeted drug resistance in cancer patients.

### Quercetin reprogramming cellular metabolism and signalling pathway in drug resistance TNBC

Metabolism disruption stands as a pivotal characteristic of cancer, driven by the rapid proliferation of cancer cells necessitating heightened energy consumption [41]. This metabolic shift not only fuels cancer growth and resistance to treatments but also influences the metabolic behavior of immune cells within the tumor microenvironment (TME) [42]. For instance, enhanced L-arginine metabolism in myeloid cells within the TME can impair lymphocyte response to tumor antigens, thereby promoting tumor growth by hindering cancer cell clearance. Consequently, the metabolic milieu surrounding cancer cells can foster their survival, progression, and metastasis [43].

In contrast to healthy cells, cancer cells primarily produce energy through glycolysis, even in environments with high oxygen concentrations- a phenomenon referred to as the Warburg effect. This metabolic adaptation enables tumor cells to generate glycolytic intermediates crucial for cell proliferation and progression. Targeting this metabolic feature, particularly in aggressive breast cancer subtypes like TNBC, presents an attractive therapeutic strategy. TNBC cells exhibit heightened glycolysis and reduced mitochondrial respiration compared to other subtypes, emphasizing their sensitivity to glycolytic inhibition. Notably, glucose transporter 1 (GLUT1), which is in charge of cancer cells' uptake of glucose, is overexpressed in breast cancer, particularly in TNBC, underscoring its role in regulating TNBC cell metabolism [44].

Phytoagents, such as EGCG, have shown promise in modulating glucose metabolism in TNBC cells by targeting key players like HIF1α and GLUT1, consequently inhibiting glycolysis [45]. Similarly, targeting enzymes involved in fatty acid synthesis, like fatty acid synthase (FASN), offers a potential avenue for sensitizing breast cancer cells to chemotherapy. Compounds like resveratrol and curcumin have demonstrated efficacy in downregulating FASN expression and lipid synthesis, thereby reducing cell survival and promoting apoptosis in breast cancer cells [46, 47].

Moreover, intracellular lipid-binding proteins, particularly FABP4 and FABP5, play crucial roles in tumor development, with elevated levels associated with poor prognosis in TNBC patients. Curcumin-mediated inhibition of the FABP5/PPARβ/δ pathway sensitizes TNBC cells to growth suppression by retinoic acid [48]. Additionally, arachidonic acid metabolic pathways contribute to TNBC carcinogenesis and metastasis, with compounds like dLGG showing promise in attenuating TNBC recurrence and metastasis by downregulating key signaling axes involving FABPs and PPARγ [49, 50].

Despite chemotherapy being the primary treatment for TNBC, acquired drug resistance remains a significant challenge. Understanding the metabolic and microenvironmental influences on drug resistance highlights the potential of phytochemicals in overcoming chemoresistance. [51] Compounds like EGCG and curcumin have shown efficacy in sensitizing TNBC cells to chemotherapy-induced cytotoxicity, offering potential strategies to combat drug resistance and improve treatment outcomes for TNBC patients.

### Mechanism of chemotherapeutic drug resistance

(A) *Increased drug efflux*—ABC transporters like MDR-1, MRP1, and BCRP, and proteins like ALDH, are often overexpressed in cancer cells, leading to drug resistance by pumping out chemotherapeutic agents [52].
(B) *Enhanced DNA repair capacity*—Proteins involved in base excision repair (BER) and nucleotide excision repair (NER) enhance DNA repair capabilities in cancer cells, contributing to resistance against DNA-damaging agents [25, 53].
(C) *Genetic and epigenetic factors*—Mutations in TP53, abnormal activation of signaling pathways (e.g., PI3K/Akt), and epigenetic changes (e.g., DNA methylation) can promote resistance by altering gene expression and cellular behavior [54].
(D) *Growth factors*—Increased levels of cytokines (e.g., IL-1, IL-6) and growth factors (e.g., eFGF) in multidrug-resistant cancer cells support survival and resistance to chemotherapy[55].
(E) *Increased metabolism of xenobiotics*—Overexpression of cytochrome P450 enzymes and phase II enzymes (e.g., GSTs, UGTs) enhance drug metabolism, reducing drug efficacy [56, 57].
(F) *Endoplasmatic reticulum (ER) stress*—ER stress triggers cellular mechanisms that promote drug resistance, including increased ROS levels, activation of NF-kB, and autophagy, which help cancer cells survive chemotherapy [58].

### Drug resistance in TNBC

**Fig. 4:**
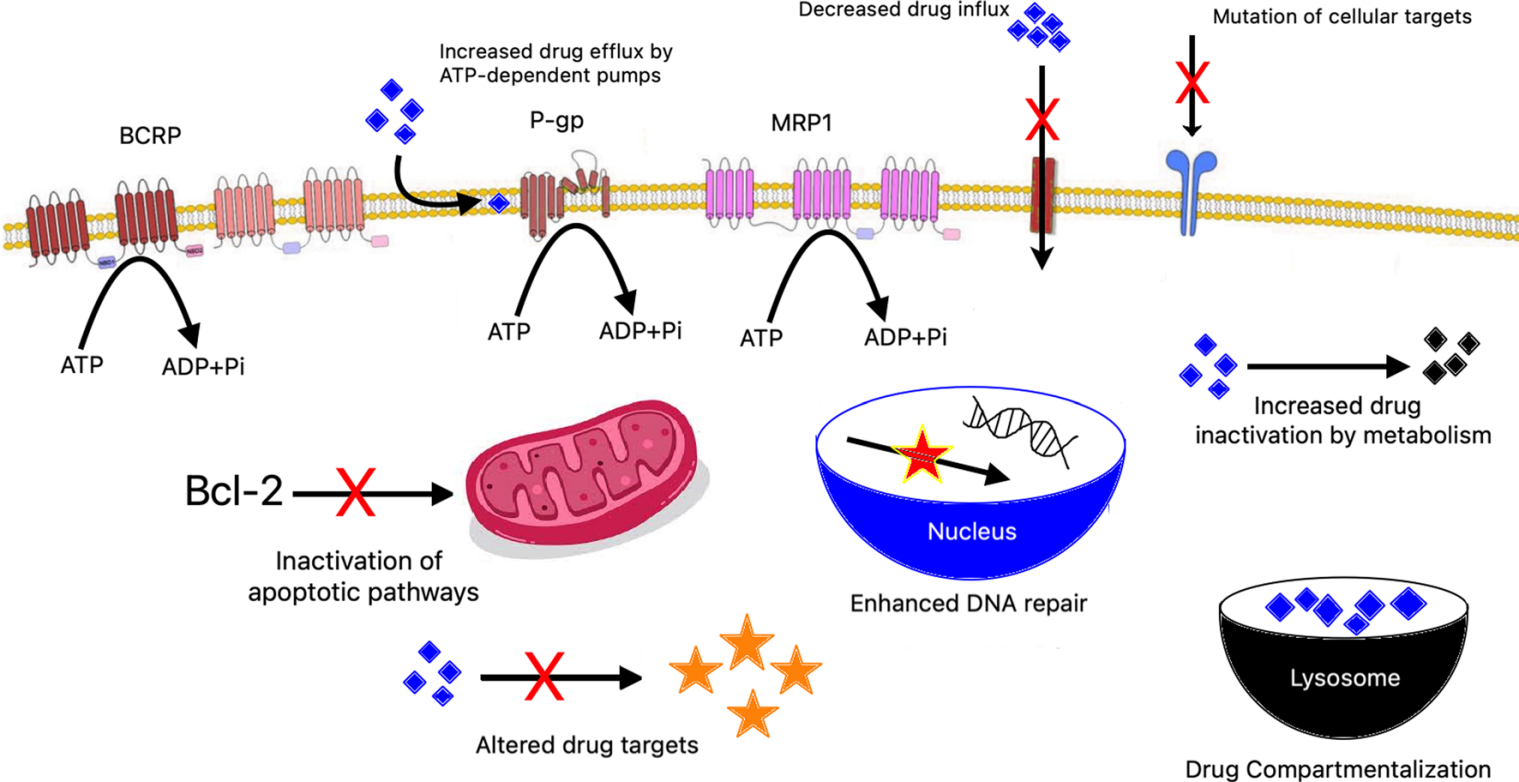
Mode of action or mechanism of resistance in chemotherapeutic drugs

Understanding the intricate interplay between tumors and the host immune system is key to unraveling the immunogenic resistance mechanisms and identifying potential intervention targets. The mechanisms contributing to drug resistance in triple-negative breast cancer (TNBC) can be summarized into three main points: weakening tumor immunogenicity, reducing antigen presentation by major histocompatibility complex (MHC), and hindering immune cell recruitment and invasion. As shown in Fig. 4. TNBC immune resistance arises from a complex interaction of many mechanisms inside the tumour environment, according to analysis of the human immune landscape [59].

However, many gaps remain in our understanding of the processes underlying medication resistance in TNBC. Firstly, TNBC lacks a standardised immune phenotyping, leading to imprecision in understanding immune resistance mechanisms [60]. Secondly, there is still a need for more research into the intricate molecular interactions occurring inside the tumour microenvironment (TME) in order to fully comprehend the influence of external variables on TNBC immune resistance. Lastly, the effectiveness of techniques to reverse immunological drug resistance is still unknown because they are mainly in the preclinical stage [61].

To address these shortcomings and advance precision therapy, several prospects are proposed:

- *Revise tumor immunophenotyping* Move beyond simplistic classifications of tumors as "cold" or "hot" immunophenotypes. Define new, standardized tumor immunophenotypes based on comprehensive immune variables [62]. This approach challenges traditional classifications and may better guide immunotherapy decisions. Examples from colorectal cancer (CRC) research demonstrate the effectiveness of such a nuanced approach, offering insights into immune escape and regulation [63].
- *Enhance understanding of external mechanisms* Enhanced comprehension of extrinsic variables causing resistance to cancer treatments, particularly the complex interactions within the TME. Investigate the role of non-immune cellular components, such as cancer-associated fibroblasts (CAFs), in shaping the immunosuppressive microenvironment [64–66]. Explore how factors like hypoxia and metabolites impact immune cell function and therapy efficacy.
- *Translate drug resistance targets to clinical practice* Develop strategies to target drug resistance mechanisms, focusing on enhancing tumor immunogenicity, increasing antigen presentation, and regulating immune effector cell recruitment. Combination immunotherapies offer promise in overcoming resistance and achieving clinical benefits. However, translating these strategies to clinical practice requires thorough clinical trials and extensive experimental designs are needed to close the gap between preclinical discoveries and practical implementations [67].

In summary, advancing our understanding of TNBC immune resistance and translating this knowledge into effective therapeutic strategies require a multifaceted approach that considers tumor heterogeneity, the tumor microenvironment, and individual immune system variability.

Nanoquercetin helps overcome chemotherapeutic drug resistance by enhancing the bioavailability, stability, and targeted delivery of quercetin. Pure quercetin suffers from poor solubility and rapid degradation, limiting its effectiveness, whereas nanoquercetin formulations, such as nanoparticles or liposomes, improve solubility and protect quercetin from degradation, resulting in higher intracellular concentrations. This enhanced delivery system facilitates better penetration and accumulation in cancer cells, effectively inhibiting drug efflux pumps like P-glycoprotein (P-gp) and ensuring higher retention of chemotherapeutic agents. Additionally, nanoquercetin provides controlled and sustained release, maintaining therapeutic levels for longer periods and continually sensitizing cancer cells to chemotherapy. Its targeted delivery capabilities concentrate the therapeutic effects at the tumor site, minimizing damage to healthy cells. Mechanistically, nanoquercetin more effectively induces apoptosis by modulating apoptotic pathways, disrupts survival pathways such as PI3K/Akt and MAPK/ERK, and reduces reactive oxygen species (ROS) levels, all of which restore cancer cells' sensitivity to chemotherapeutic drugs. Thus, nanoquercetin significantly enhances the therapeutic potential of quercetin, making it a powerful tool in combating drug-resistant cancer cells.

### Quercetin enhance effectiveness of conventional chemotherapeutic agents

The resistance or insensitivity of cancer cells to chemotherapeutic agents poses a significant challenge in cancer treatment. On the other hand, current studies indicate that nano-quercetin may improve the chemotherapeutic drugs' sensitivity and effectiveness. Numerous flavonols, such as morin, kaempferol, and quercetin, have shown strong effects on cancer cell chemoresistance.

Abnormal activation of androgen receptor (AR) and PI3K/Akt signaling pathways is implicated in docetaxel resistance. Quercetin has shown promising effects in reversing docetaxel resistance in prostate cancer cells (LNCaP/R, PC-3/R) both in vitro and in vivo [68]. It was discovered to deactivate the androgen receptor and PI3K/Akt signalling pathways, reverse the overexpression of P-glycoprotein (P-gp), and prevent the phenotypic development of stem-like and mesenchymal cells [69]. Furthermore, quercetin and docetaxel combination therapy effectively reduced proliferation in vivo and slowed the growth of tumours.

By inducing reactive oxygen species (ROS) and endoplasmic reticulum (ER) stress in PC-3 prostate cancer cells, quercetin has also been shown to enhance the therapeutic efficiency of paclitaxel in vitro [70]. Benefits of this combined treatment were also seen in a mouse model with PC-3 tumours.

In pancreatic cancer cells (MIA Paca-2 and MIA Paca-2 GEM-resistant), quercetin increased cell death and gemcitabine sensitivity by specifically inhibiting the receptor for advanced glycation end products (RAGE)/PI3K/AKT/mTOR axis [71].

Additionally, Quercetin has been shown to reverse multidrug resistance (MDR) in breast cancer cells (MCF-7 and MCF-7/Dox) by nuclear translocation of Y-box binding protein 1 (YB-1), upregulating P-gp expression and killing cancer stem cells (CSCs) [72].

All things considered, our results point to the possibility of using nano-quercetin as an adjuvant therapy to boost the efficiency of traditional chemotherapeutic drugs and get around drug resistance in different kinds of cancer [73].

A thorough summary of the particular mechanisms by which quercetin improves the therapeutic efficiency of traditional chemotherapeutic drugs may be found in Table 3. These findings point to a substantial potential for improved treatment efficacy when quercetin and traditional chemotherapeutic drugs are combined.

**Table 3:** Quercetin enhancing the efficacy of conventional chemotherapeutic agents [74–77]

Quercetin + Drugs	Study details and mechanism
Quercetin (+ Docetaxel)	In prostate cancer models resistant to docetaxel, combining quercetin reversed drug resistance, slowed tumor growth, and inhibited proliferation by suppressing P-gp upregulation, mesenchymal and stem-like cell phenotypes, and androgen receptor and PI3K/Akt signaling pathways
Quercetin (+ Paclitaxel)	Quercetin enhanced the therapeutic effectiveness of paclitaxel in prostate cancer cells and a murine model by inducing ER stress and generating ROS, leading to reduced cell proliferation and migration, increased apoptosis, and arrest of the cell cycle at the G2/M phase
Quercetin (+ Gemcitabine)	In pancreatic cancer cells resistant to gemcitabine, quercetin increased sensitivity to the drug and promoted cell death by modulating the RAGE/PI3K/AKT/mTOR axis, primarily through RAGE inhibition
Quercetin (+ Doxorubicin/paclitaxel/vincristine)	When combined with doxorubicin, paclitaxel, and vincristine in breast cancer cells resistant to these drugs, quercetin enhanced their activity by reversing multi-drug resistance through downregulation of P-gp and elimination of cancer stem cells via YB-1 nuclear translocation

### Nanotechnologic approches to facilitate quercetin‑conducted chemotherapeutic anti‑cancer toxicity

The utilization of nanotechnology presents a promising approach to enhance the interaction between quercetin and chemotherapy, overcoming limitations such as poor solubility and bioavailability. Studies have demonstrated that polymeric micelles loaded with quercetin can effectively inhibit multidrug resistance (MDR) in cancer cells by interfering with P-glycoprotein (P-gp) efflux and mitochondrial membrane potential. Furthermore, studies have demonstrated enhanced intracellular absorption and cytotoxicity against breast cancer cells when quercetin is co-encapsulated in mixed polymeric micelles alongside traditional chemotherapeutic drugs like paclitaxel and doxorubicin [17].

In addition, the creation of double-targeted nanocarriers, like QDAF (Quercetin-3-3-dithiodipropionic acid-Astragalus polysaccharides-Folic acid), has been effective in decreasing MDR in breast cancers that express the oestrogen receptor Î ± (ERÎ ±) [35]. Known as "nano-pomegranates," these nano-targeted delivery systems boost cellular uptake, apoptosis, and necrosis in cancer cells in vitro and show reduced systemic toxicity and enhanced anticancer activity in vivo. Overall, co-administration of flavonoids with conventional chemotherapy drugs like quercetin in nano-carrier systems holds promise for enhancing chemotherapeutic efficacy, sensitizing cancer cells to treatment, inhibiting chemoresistance, and reducing cytotoxicity in healthy tissues [75].

### Current challenges and future aspects of nanoquercetin against TNBC therapy

Throughout history, natural products derived from plants have served as a cornerstone in the discovery of pharmaceuticals. It's estimated that a significant portion of today's medical treatments originated from these plant-derived compounds. Examples like paclitaxel, camptothecin, and etoposide illustrate the widespread use of plant-based chemotherapy drugs. However, despite their efficacy, these anticancer medications often encounter resistance and adverse effects. Consequently, current research is focused on developing modern pharmacotherapies to complement existing treatments [78] This urgency is particularly evident in addressing triple-negative breast cancer (TNBC), known for its aggressiveness and challenges in treatment. Recent strategies targeting novel pathways aim to improve therapeutic outcomes by addressing chemoresistance and TNBC recurrence. Combining plant-derived compounds with conventional therapies has shown promise in enhancing efficacy and reducing adverse effects in preclinical studies.^82^ Notably, certain natural products like curcumin and mistletoe are recognized as "Integrative, Alternative, and Complementary Therapies" by the National Cancer Institute. Moreover, ongoing clinical trials explore the potential benefits of other plant-derived compounds, such as sulforaphane and peppermint essential oil, in managing symptoms and side effects in breast cancer patients. However, further validation through rigorous clinical trials is necessary to confirm their efficacy for TNBC. Adopting systematic approaches to identify and characterize bioactive plant-derived compounds holds immense potential in addressing the challenges posed by TNBC, representing a crucial goal in advancing treatment options for this formidable cancer.

## Conclusion and future prospective

The pharmaceutical research field has seen significant advancements in delivery technologies aimed at overcoming formulation challenges and enhancing the administration of various drug moieties. These delivery systems not only improve the pharmacological and therapeutic characteristics of loaded moieties but also offer controlled drug distribution, leading to longer-lasting, safer, and more effective effects in the body. Nanotechnology-based formulations, in particular, have garnered significant interest in the research community for administering dermatology products, cosmetics, nutraceuticals, and pharmaceuticals. These formulations offer benefits such as ex-vivo cutaneous drug deposition, enhanced skin permeation, and improved therapeutic efficacy of bioactives. While many innovative nano carrier systems have been investigated for topical administration, effective administration still poses challenges. Recent studies have proposed the use of transfersome dispersion for topical delivery of quercetin to enhance bone formation, and nanoemulsions have shown promise in overcoming solubility limitations, achieving target specificity, and enabling modified release. Through chemical structural modification or coupling with another moiety, nanoformulations coupled with gels also demonstrate good delivery qualities ideal for topical administration, offering targeted and controlled drug delivery. Additionally, by improving kinetic saturation solubility and concentration gradient, nanocrystals have proven to be an effective formulation approach for boosting cutaneous bioavailability of less soluble moieties. Despite these advancements, there is ongoing exploration of quercetin in various advanced drug delivery systems, indicating the continuous efforts to optimize its delivery and therapeutic potential.

The cosmeceutical and pharmaceutical industries are highly promising for plant-based bioactive chemicals like quercetin because of its potential therapeutic uses in a range of areas, including as UV protection, skin regeneration, moisturising, anti-aging, and disease prevention connected to the skin. Quercetin, in particular, has garnered attention for its wide-ranging physiological and health-promoting effects, making it a valuable ingredient in pharmaceuticals, nutraceuticals, and cosmetics [79].

The emergence of nanotechnology-based drug delivery systems has provided a solution to the limitations of conventional delivery methods. Nanoformulations offer the advantage of targeted delivery, improved stability, and enhanced bioavailability of quercetin. While numerous nanoformulations for topical delivery of quercetin have been developed, their translation into clinical applications has been limited [80].

The clinical efficacy of nanoformulations is crucial for their successful translation into clinical practice. Demonstrating improved biological effects and therapeutic outcomes compared to conventional formulations is essential for gaining regulatory approval and commercial viability. The market for nano goods using naturally derived components is anticipated to grow over the next ten years, replacing synthetic chemicals as the usage of herbal nanomedicine and nano-drug delivery systems continues to gain popularity. Nanoquercetin, an advanced formulation of the flavonoid quercetin, enhances cancer treatment by improving bioavailability and stability, enabling targeted delivery to tumor cells, and overcoming drug resistance [81]. It retains quercetin's antioxidant, anti-inflammatory, and anti-proliferative effects, which can complement conventional therapies and reduce their side effects. This targeted and effective approach may improve patient compliance and outcomes, and aligns with personalized medicine principles, offering a promising strategy for cancer and potentially other conditions associated with inflammation and oxidative stress.

However, to realize the full potential of nano-based drug delivery for quercetin and other plant-based bioactive compounds, further comprehensive research is needed to optimize formulations, evaluate safety profiles, and establish clinical efficacy. Collaboration between researchers, clinicians, and industry stakeholders will be essential for advancing the development and commercialization of nanoformulations in the pharmaceutical and cosmeceutical sectors.

## Data Availability

No datasets were generated or analysed during the current study.

## Abbreviations

APIs: Active pharmaceutical ingredients
ADME(T): Absorption, distribution, metabolism, excretion, and toxicology
CHSNPs: Chitosan nanoparticles
EMT: Epithelial-mesenchymal transition
ALDH: Aldehyde dehydrogenase
ABC: ATP-binding cassette
MDR: Multidrug resistance
TKIs: Tyrosine kinase inhibitors
TME: Tumor microenvironment
GLUT1: Glucose transporter 1
TNBC: Triple negative breast cancer
FASN: Fatty acid synthase
CSCs: Cancer stem cells
BCRP: Breast cancer resistance protein
NER: Nucleotide excision repair
BER: Base excision repair
CYPs: Cytochrome P450 enzymes
RAGE: Receptor for advanced glycation end products
PARP1: Poly(ADP-ribose) polymerase 1
